# Experimental datasets on properties of river sand as an aggregate in replacement of crushed rock for interlocking stones production

**DOI:** 10.1016/j.dib.2018.08.056

**Published:** 2018-08-28

**Authors:** Adekunle M. Ajao, Babatunde F. Ogunbayo, Kunle E. Ogundipe, Opeyemi Joshua, Oluwarotimi M. Olofinnade

**Affiliations:** a Department of Building Technology, Covenant University, Ota, Nigeria; b Department of Civil Engineering, Covenant University, Ota, Nigeria

**Keywords:** River sand, Crushed stone as fine aggregate, Cement, Compressive strength, Experimental procedures

## Abstract

The data explored the assessment of the quality of river sand as an aggregate in replacement of crushed stones which are widely used by majority of manufacturers in production of interlocking stones. Experimental tests carried out on river sand and crushed rock as aggregates include: Grain size distribution, Specific gravity, moisture content determination and Bulk density to determine the quality behavior parameters and (compressive strength) to determine the strength parameters. The data of the experiments are presented in Tables and Bar charts.

## Specification Table

**Table:** 

Subject area	Building Construction, Building Materials Science
More specific subject area	Building Materials Development
Type of data	Table, Figure
How data was acquired	The data were obtained through experimental tests and procedures under conducive atmospheric condition in the laboratory and simple statistical tools were employed for the analyses.
Data format	Raw data obtained were processed and analysed.
Experimental factors	Various tests on Physical properties and strength parameters of aggregate samples such as moisture content, Bulk Density, Specific gravity, Sieve Analysis and compressive strength were carried out.
Experimental features	Engineering properties of River Sand and Crushed Rock with various laboratory tests.
Data source location	Ota, Atan, Ado-odo Local Government Area, Ogun State, Nigeria.
Data accessibility	The data is available with the article

## Value of the data

• The data can be used as a clear indication for finding result comparison from other countries where the use of the said materials is prevalent.

• The data can be adopted for governmental policy on low-cost housing scheme production for the benefit of low-income earners.

• The data provided conducive room for further studies on the reliability of local building materials in the building industries.
• The data provided detailed experimental procedures on how river sand could be used instead of crushed stone thereby reducing its production cost.

## 1 Data

**Fig. 1:**
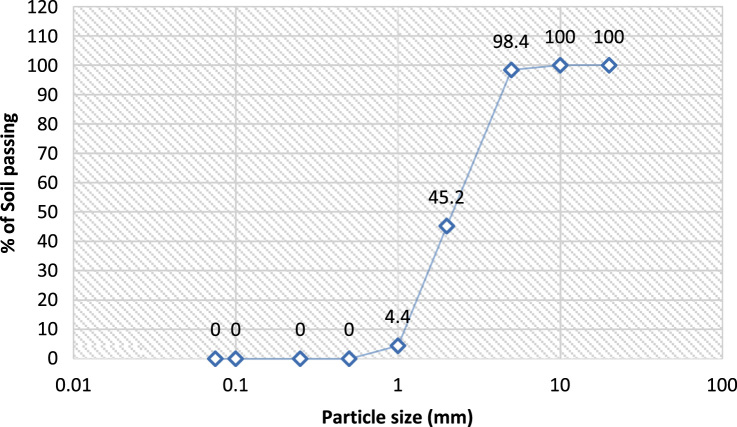
Sieve analysis of 100% river sand.

**Fig. 2:**
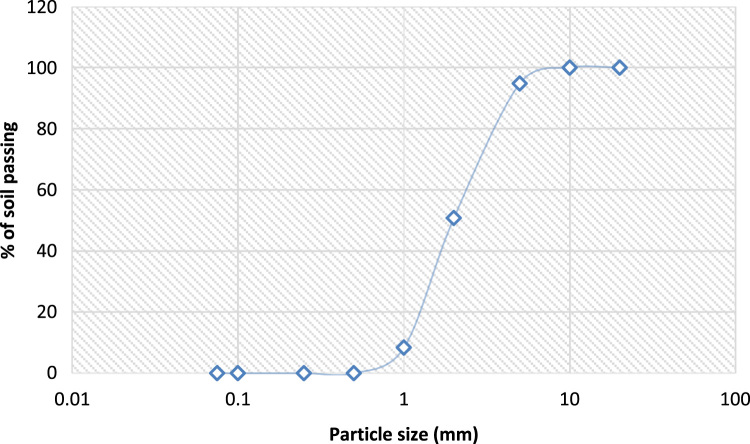
Sieve analysis of 50%:50% rivers sand and crushed stone.

**Fig. 3:**
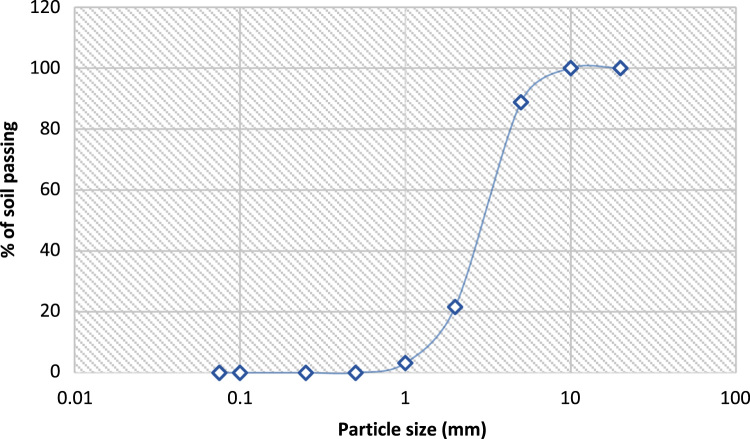
Sieve analysis of 100% crushed rock.

The data assessed the usefulness of available river sand in replacement of crushed stones in the production of interlocking stone. Related articles are [1], [2], [3], [4]. The data presented in Table 1, Table 2, Table 3, Table 4, Table 5, Table 6, Table 7, Table 8, Table 9 were obtained from the analyses of property parameters of river sand and crushed rock to determine its suitability for construction activities. The behaviour of 100% RS, 50%/50% RS/CR, 100% CR as indicated in Table 1, Table 2, Table 3, Table 4, Table 5, Table 6, Table 7, Table 8, Table 9 illustrated that all the specimens met required standards but River sand had the highest value [5], [6], [7], [8], [9], [10] The variance in the value of aggregates in moisture content determination, specific gravity and bulk density determination were equally illustrated in the tables. Data of grading sizes parameters are shown in Fig. 1, Fig. 2, Fig. 3 and they were all in conformity with the standard requirements [8], [9], [10].

**Table 1:** Moisture content determination of 100% river sand.

**Tin no**	**1A (g)**	**1B (g)**
**Tin + Wet Soil**	68	80
**Tin + Dry Soil**	67	79
**Weight of Tin**	34	40
**Weight of Water**	1.0	1.0
**Weight of dry soil**	33	39
**M.C. %**	**3.00**	**2.60**
	Average: - **2.80**

**Table 2:** Moisture content determination of 50%:50% (river sand & crushed rock).

**Tin no**	**2A (g)**	**2B (g)**
**Tin + Wet Soil**	70	80
**Tin + Dry Soil**	68	79
**Weight of Tin**	34	40
**Weight of Water**	1.0	1.0
**Weight of dry soil**	34	39
**M.C. %**	**5.90**	**2.56**
	Average: - **4.23**

**Table 3:** Moisture content determination of 100% crushed rock.

**Tin no**	**3A (g)**	**3B (g)**
**Tin + Wet Soil**	60	81
**Tin + Dry Soil**	68	79
**Weight of Tin**	34	40
**Weight of Water**	1.0	1.0
**Weight of dry soil**	33	39
**M.C. %**	**5.88**	**5.13**
	Average: - **5.51**

**Table 4:** Specific gravity of 100% river sand.

Determination number-	1A	1B
**Mass of Empty Pycnometer (g)**	**170**	**180**
**Mass of Empty Pycnometer + Sample (g)**	270	280
**Mass of Empty Pycnometer + Sample + Water (g)**	526	536
**Mass of Sample (g)**	100	100
**Mass of Pycnometer + Water**	463	474
**Mass of Sample in Water (g)**	356	356
**Volume of Pycnometer (cm**^**3**^**)**	290.9	290.9
**Specific Gravity**	**2.70**	**2.63**
	Average**: 2.67**	
	Specification: 2.60–2.72

**Table 5:** Specific gravity of 50%:50% (river sand & crushed rock).

Determination number-	2A	2B
**Mass of Empty Pycnometer (g)**	**170**	**180**
**Mass of Empty Pycnometer + Sample (g)**	250	260
**Mass of Empty Pycnometer + Sample + Water (g)**	507	516
**Mass of Sample (g)**	80	80
**Mass of Pycnometer + Water**	458	466
**Mass of Sample in Water (g)**	337	336
**Volume of Pycnometer (cm**^**3**^**)**	290.9	290.9
**Specific Gravity**	**2.58**	**2.66**
	Average**: 2.62**	
	Specification: 2.60–2.72

**Table 6:** Specific gravity of 100% crushed rock.

Determination number-	3A	3B
**Mass of Empty Pycnometer (g)**	**170**	**180**
**Mass of Empty Pycnometer + Sample (g)**	250	260
**Mass of Empty Pycnometer + Sample + Water (g)**	508	518
**Mass of Sample (g)**	80	80
**Mass of Pycnometer + Water**	460	468
**Mass of Sample in Water (g)**	336	336
**Volume of Pycnometer (cm**^**3**^**)**	290.9	290.9
**Specific Gravity**	**2.50**	**2.67**
	Average**: 2.59**	
	Specification: 2.60–2.72

**Table 7:** Bulk density 100% river sand.

Determination number-	1A	1B
**Weight of Density Container (g)**	**1840**	**1840**
**Percentage of water added (%)**	4.000	4.000
**Weight of Sample (g)**	1736	1680
**Weight of Container + Sample + Water (g)**	3576	3520
**Volume of Density Container (cm^3^)**	944	944
**Bulk Density**	**1.84**	**1.78**
	Average: **- 1.81**	
	Specification: - > 1.3

**Table 8:** Bulk density of 50%:50% (river sand & crushed rock).

Determination number-	3A	3B
**Weight of Density Container (g)**	**1840**	**1840**
**Percentage of water added (%)**	4.000	4.000
**Weight of Sample (g)**	1686	1590
**Weight of Container + Sample + Water (g)**	3526	3430
**Volume of Density Container (cm^3^)**	944	944
**Bulk Density**	**1.79**	**1.68**
	Average: **- 1.74**	
	Specification: - > 1.3

**Table 9:** Bulk density of 100% crushed rock.

Determination number-	2A	2B
**Weight of Density Container (g)**	**1840**	**1840**
**Percentage of water added (%)**	4.000	4.000
**Weight of Sample (g)**	1646	1580
**Weight of Container + Sample + Water (g)**	3486	3420
**Volume of Density Container (cm^3^)**	944	944
**Bulk Density**	**1.74**	**1.67**
	Average: **- 1.71**	
	Specification: - > 1.3

## 2 Experimental design, materials and methods

The specimens of fine aggregate used for this data were obtained from Ota and Atan Tipper garage, Ado-odo Local Government Area, Ogun State, Nigeria. The River sand (RS) and crushed Rock (CR) used were; (100%RS), (100%CR) and (50%RS: 50%CR). Ordinary Portland cement (OPC) grade 42.5N was used and it was supplied in good condition. Portable water used for the study conformed to required standard [11]. The experimental procedures were carried out in the following order: 72 interlocking concrete cubes were produced under controlled temperature with ratio 1:3 and 1:4 respectively and it was cured through immersion method. Compressive strength of concrete cubes was determined after curing for 7 days, 14 days, 21 days and 28 days respectively. To provide a good justification for the test results, several tests such as grain size distribution, specific gravity, moisture content determination and bulk density were conducted on the samples to determine its physical properties and suitability. However, various experimental procedures conducted on engineering properties of river sand and crushed rock were in conformity with the recommended standards [5].

**Fig. 4:**
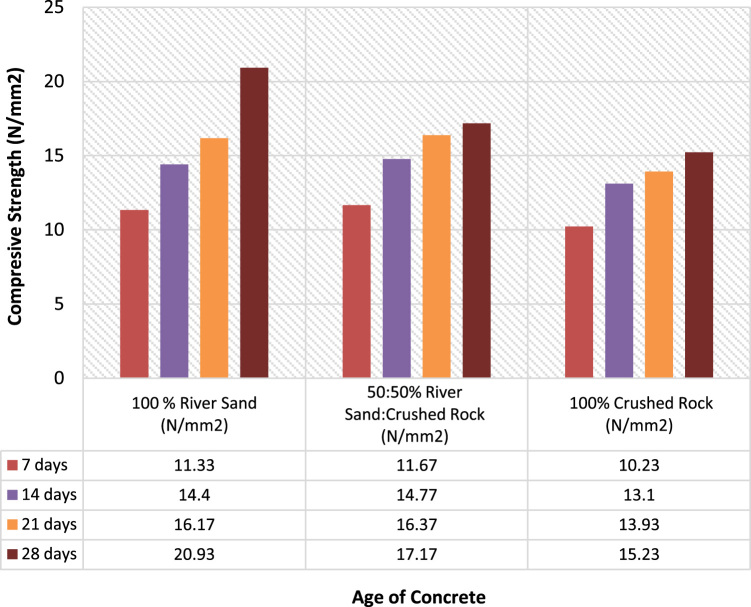
Compressive strength of the soil for 1:3.

**Fig. 5:**
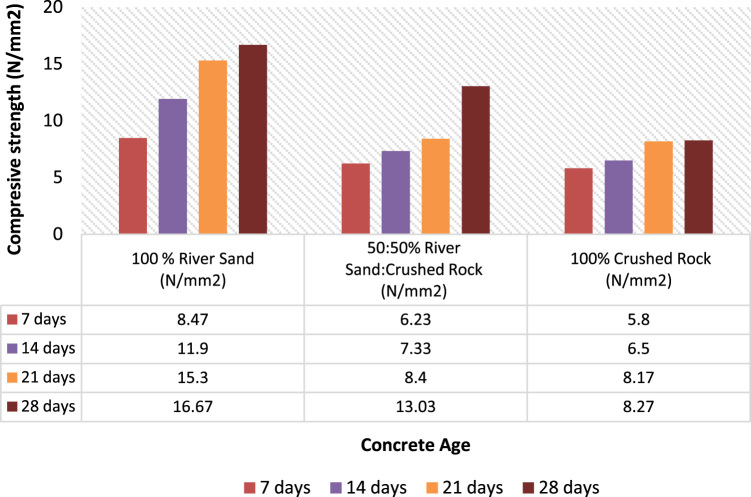
Compressive strength of the soil for 1:4.

The results of compressive strength for the three samples are shown in Figs. 4 and 5 and methods for mixing, curing, and strength test parameter were strictly followed and they were all in accordance to the standards [12], [13], [14], [15], [16], [17], [18], [19]. Figs. 4 and 5 showed differences in strength parameters of the samples used. Thereby, the River sand had the highest compressive strength value with ratio (1:3) over Crushed rock which is most widely used by the interlocking stones manufacturers with assumption of colour resemblance to ordinary Portland cement. The data presented on river sand is a proof to be cost effective when compared with previous studies on crushed rock [1], [2], [3], [4]. The outcome of the strength test revealed the performance and standard of local building materials in low cost housing production [19], [20]. The presentation of data is also similar to that of [21], the experimental procedure of data presented took into consideration the recommendations of [22], [23], [24].
